# A Kalman Filtering Algorithm for Measurement Interruption Based on Polynomial Interpolation and Taylor Expansion

**DOI:** 10.3390/e26030243

**Published:** 2024-03-10

**Authors:** Jianhua Cheng, Zili Wang, Bing Qi, He Wang

**Affiliations:** College of Intelligent Systems Science and Engineering, Harbin Engineering University, Harbin 150001, China; ins_cheng@163.com (J.C.); wangzili@hrbeu.edu.cn (Z.W.); wang_he@hrbeu.edu.cn (H.W.)

**Keywords:** measurement interruption, polynomial fitting, Taylor expansion, virtual measurement, adaptive filtering

## Abstract

Combined SINS/GPS navigation systems have been widely used. However, when the traditional combined SINS/GPS navigation system travels between tall buildings, in the shade of trees, or through tunnels, the GPS encounters frequent signal blocking, which leads to the interruption of GPS signals, and as a result, the combined SINS/GPS-based navigation method degenerates into a pure inertial guidance system, which will lead to the accumulation of navigation errors. In this paper, an adaptive Kalman filtering algorithm based on polynomial fitting and a Taylor expansion is proposed. Through the navigation information output from the inertial guidance system, the polynomial interpolation method is used to construct the velocity equation and position equation of the carrier, and then the Taylor expansion is used to construct the virtual measurement at the moment of the GPS signal interruption, which can make up for the impact of the lack of measurement information on the combined SINS/GPS navigation system when the GPS signal is interrupted. The results of computer simulation experiments and road measurement tests based on the loosely combined SINS/GPS navigation system show that when the carrier faces a GPS signal interruption situation, compared with a combined SINS/GPS navigation algorithm that does not take any rescue measures, our proposed combined SINS/GPS navigation algorithm possesses a higher accuracy in the attitude angle estimation, a higher accuracy in the velocity estimation, and a higher accuracy in the positional localization, and the system possesses higher stability.

## 1. Introduction

Due to its many uses in positioning and attitude determination, the loosely integrated SINS/GPS navigation system has seen significant adoption in the military and commercial sectors in recent years [1,2,3]. An independent navigation system that does not rely on outside data is the Strapdown Inertial Navigation System (SINS). Its high anti-jamming ability and good camouflage are its main advantages, but, over time, navigation errors tend to mount. The Global Positioning System (GPS) has the ability to give customers continuous, three-dimensional position and velocity data in all weather conditions. However, it has drawbacks such as low dynamic performance, vulnerability to electromagnetic interference, and easily occluded satellite signals [4,5,6]. Combining the SINS and GPS can improve performance over utilizing either navigation sensor alone by addressing the drawbacks of the SINS’s low long-term accuracy and the GPS’s poor dynamic performance. As of right now, the most popular navigation mode for land vehicle navigation systems is the integrated SINS/GPS navigation system, which can meet real-time positioning needs for a long time with high accuracy and low error output [7,8,9]. In certain difficult settings, the integrated SINS/GPS navigation system is still inadequate. For instance, GPSs may not provide accurate navigational data due to signal obstruction brought on by structures like buildings, bridges, and trees in urban areas, as well as multipath effects in tunnels and canyons. Long-term GPS rejection will cause the integrated system to deteriorate into a purely inertial navigation system, which will lead to the rapid accumulation of navigation errors [10].

Loosely combined SINS/GPS navigation systems are generally fused using the Kalman filter algorithm, and the solution of the Kalman filter is reliable only with a reliable carrier motion function model, a noisy stochastic model, and a reasonable state estimation method. In the real measurement environment, it is generally difficult to ensure the regular motion of the motion carriers, so the construction of the exact function model is very difficult. The acquisition of a priori information for stochastic models is generally based on pre-test statistical information, which is also different from the actual situation. Therefore, how to utilize the current observation information and state valuation to update the a priori information and compensate for the motion equation error has become a hot issue in dynamic navigation and positioning research.

The SINS/GPS pine combination navigation system uses the speed provided by the GPS and position as the observation. However, signal blockage caused by structures such as bridges and trees in urban environments and multipath effects in canyons and tunnels can cause interruptions in the output of the GPS device, thus preventing the acquisition of effective navigation information. At this time, if the measurement information is ignored and only pure inertial guidance is relied on, the loosely combined SINS/GPS navigation system will have a large error or even lead to the dispersion of the navigation results. Therefore, when designing a combined SINS/GPS navigation system, it is essential to consider how to minimize the significant impact of GPS signal interruptions on the navigation system.

A GPS signal interruption will definitely lead to a significant reduction in the positional accuracy of the combined navigation system and the estimation accuracy of the related navigation parameters. At present, there are two main improvement methods. One is to construct an integrated navigation filtering equation with motion constraints (called internal constraints) according to the inherent motion law of the carrier. The other is to utilize the observation information from redundant sensors to construct a filter equation with additional observation constraints (called external constraints).

In terms of internal constraints, a series of research works have been carried out by many international scholars to address the problem of the dispersion of vehicle combination navigation results due to a GPS signal interruption. According to the principle of kinematics, when the vehicle moves on the ground, if there is no lateral sliding or up-and-down jumping fluctuation, the velocity of its lateral and vertical components will be close to zero, i.e., the non-holonomic constraint (NHC) of the vehicle motion, so the virtual velocity observables can be constructed in combined navigation. In relevant studies, scholars have proposed some non-integrity constraint theories and carried out experimental verification on the basis of the velocity non-integrity constraint method, the position non-integrity constraint method, and attitude non-integrity constraint method. In [11], Benz proposed a non-complete velocity constraint method, assuming a lateral velocity of 0 for each wheel, and experiments showed that it was effective in improving the average localization error with a GPS interruption of 90 s. In [12], Ren proposed a method that used non-complete elevation constraints to mitigate low-frequency multipath and conducted GPS deformation detection experiments in urban environments, which showed that the root-mean-square (RMS) values of the navigation parameters using this method were smaller and improved the positioning accuracy. In [13], Klein proposed an attitude angle velocity constraint method for urban occlusion environments by setting the value of the horizontal attitude angle to zero, and experiments showed that the navigation accuracy was effectively improved. Niu proposed heading angular velocity constraints in the literature [14], which can improve the low dynamic vehicle navigation accuracy, and analyzed the observability of non-complete constrained observations, providing different strategies for selecting observations for different constraints.

In terms of external constraints, a large number of scholars in China and abroad have carried out a series of important and effective research work to construct additional observation constraint equations by utilizing the observation information from redundant sensors and, ultimately, to realize the purpose of improving the accuracy of the navigation parameter estimation of combined navigation systems. Xiang proposed a combined SINS/GNSS/2D-LDV navigation system with an asynchronous Kalman filter in the literature [15], and experiments showed that a high positioning accuracy could still be maintained by introducing external measurements in the case of frequent GPS signal failures. In [16], Lyu designed an information fusion technique based on an adaptive sharing factor by combining SINS, GPS, and VDM multi-source sensors to establish the VDM observation information in the process of vehicle turning. The algorithm effectively improved the positioning accuracy while taking into account the reliability and fault tolerance of the system. Hu added external stereo-vision measurements to the literature [17], and experiments showed that this design scheme could significantly improve the system’s localization accuracy during frequent GPS failures. Liu proposed a tightly combined GPS/UWB/VIO-based navigation algorithm in [18], which improved the horizontal positioning performance of the system in GPS-obscured outdoor and indoor environments with the help of external gauging information.

In summary, constraint methods function to both reduce the estimation error of navigation-system-related parameters and improve the reliability of the system. Kalman filter, anti-differential filter, and adaptive filter models with constraints have already been used in corresponding theoretical and computational methods; in addition, parameter estimation and its nature with internal and external constraints have also been discussed in papers. However, the actual performance of combined navigation for specific approximate motion constraints has not been sufficiently analyzed.

This study focuses on the high-dimensional loosely coupled integrated SINS/GPS navigation system facing a harsh and complex environment with interrupted measurement information. Based on polynomial interpolation and Taylor expansion theory, this paper constructs a virtual measurement and proposes a solution. The main innovations and contributions of this paper are as follows:

1. In order to deal with the problem of missing measurement information caused by GPS signal interruptions that may exist in the system, we use the navigation information output from the inertial guidance system to construct the velocity equation and position equation of the carrier by the polynomial interpolation method. Then, during the GPS signal interruption, we construct a virtual measurement by a Taylor expansion so as to make up for the impact of the missing measurement information on the navigation system when the GPS signal is interrupted, and finally, we improve the stability, reliability, and accuracy of the system.
2. In this study, a computer simulation experiment and a real road measurement experiment were performed. The computer simulation experiment is divided into two parts, which are the combined navigation experiment when the measurement is interrupted in the segmented motion state and the combined navigation experiment when the measurement is interrupted in the full-motion state. The real road test experiment was chosen to be conducted in an urban environment.
3. Scientifically and accurately evaluating the performance of various algorithms in the system is an important issue. We use the magnitude of the average root-mean-square error (ARMSE) of the carrier’s attitude, velocity, and position as a judgment criterion in this paper. We also give a table of the ARMSEs for all the algorithms under road test conditions, and the experimental conclusions presented at the end of the paper.

The remainder of this article is organized in the following order. Section 2 gives the solution process of the classical Kalman filtering algorithm, polynomial interpolation theory, Taylor expansion theory, and the solution process of our proposed PIT-AKF algorithm. Section 3 designs simulation experiments and real road tests based on the algorithm and experimental model proposed in Section 2 and gives the test results of the experiments. Section 4 analyzes and discusses the test results of the experiments. We give the experimental conclusion in Section 5 that our proposed PIT-AKF solution is more effective when the navigation system faces GPS signal interruption interference, and not only are the system’s navigation parameter accuracy and positioning accuracy improved, but the stability of the navigation system is also strengthened.

## 2. Materials and Methods

### 2.1. An Adaptive Kalman Filtering Algorithm for Measurement Interruption Based on Polynomial Interpolation and Taylor Expansion (PIT-AKF)

The solution process of the discrete standard-type Kalman filtering algorithm can be expressed as shown in the following equations [19,20,21,22]:

(1) $$X_{k}=\Phi_{k,k-1}X_{k-1}+W_{k-1},$$

(2) $$Z_{k}=H_{k}X_{k}+\xi_{k},$$

In Equations (1) and (2), $X_{k}$ denotes the state estimate at moment *k*, $\Phi_{k,k-1}$ denotes the one-step transfer matrix from moment k−1 to moment *k*, $W_{k-1}$ denotes the noise sequence of the system, $Z_{k}$ denotes the measurement value at moment *k*, $H_{k}$ denotes the measurement matrix, and $\xi_{k}$ denotes the measurement noise sequence.

One-step prediction is used to predict the state at moment *k* based on the estimation of the state at moment *k* − 1, i.e., to make a linear minimum variance estimation of $X_{k}$ based on the quantitative measurements $Z_{k-1}$ at moment *k* − 1. Then,

(3) $${\hat{X}}_{k/k-1}=E*\left[X_{k}/Z_{1},Z_{2},\cdots ,Z_{k-1}\right]=E*\left[\left(\Phi_{k,k-1}X_{k-1}+W_{k-1}\right)/Z_{1},Z_{2},\cdots ,Z_{k-1}\right],$$

which is expanded to obtain

(4) $${\hat{X}}_{k/k-1}=\Phi_{k,k-1}{\hat{X}}_{k-1},$$

A one-step prediction of the value ${\hat{X}}_{k/k-1}$ and the real state $X_{k}$ will produce a certain amount of error; the error can be expressed as

(5) $${\tilde{X}}_{k/k-1}=X_{k}-{\hat{X}}_{k/k-1},$$

which is expanded to obtain

(6) $${\tilde{X}}_{k/k-1}=X_{k}-{\hat{X}}_{k/k-1}=\Phi_{k,k-1}{\tilde{X}}_{k-1}+W_{k-1},$$

Then, the one-step prediction mean-square-error array $P_{k/k-1}$ can be expressed as

(7) $$P_{k/k-1}=E\left[{\tilde{X}}_{k/k-1}{\tilde{X}}_{k/k-1}^{T}\right],$$

Substituting Equation (6) into Equation (7), the expansion yields

(8) $$P_{k/k-1}=\Phi_{k,k-1}P_{k-1}\Phi_{k,k-1}^{T}+Q_{k-1},$$

The gain matrix $K_{k}$ is chosen with the criterion of minimizing the estimated mean-square-error array $P_{k}$, which has the following form:

(9) $$P_{k}=E\left[{\tilde{X}}_{k}{\tilde{X}}_{k}^{T}\right],$$

again, because

(10) $${\tilde{X}}_{k}=X_{k}-{\hat{X}}_{k}=X_{k}-\left[{\hat{X}}_{k/k-1}+K_{k}\left(Z_{k}-H_{k}{\hat{X}}_{k/k-1}\right)\right],$$

Substituting Equation (10) into Equation (9) and expanding it, according to the principle of extreme values, it can be deduced that

(11) $$K_{k}=P_{k/k-1}H_{k}^{T}{\left(H_{k}P_{k/k-1}H_{k}^{T}+R_{k}\right)}^{-1},$$

The substitution of the measured one-step prediction $H_{k}{\hat{X}}_{k|k-1}$ for the true measured value $Z_{k}$ will also cause a certain error, which can be expressed as follows:

(12) $${\tilde{Z}}_{k|k-1}=Z_{k}-H_{k}{\hat{X}}_{k|k-1}=H_{k}{\tilde{X}}_{k/k-1}+\xi_{k},$$

where ${\hat{X}}_{k|k-1}$ denotes the one-step prediction value of the state, and in filter theory, ${\tilde{Z}}_{k|k-1}$ is called the new interest, which is denoted by $v_{k}$. From the above Equation (12), it can be seen that the residuals contain information about the one-step prediction error, and the appropriate weighting of ${\tilde{Z}}_{k|k-1}$ can separate ${\tilde{X}}_{k/k-1}$ and be used to correct ${\hat{X}}_{k|k-1}$, which can provide the estimation of the state:

(13) $${\hat{X}}_{k}={\hat{X}}_{k/k-1}+K_{k}\left(Z_{k}-H_{k}{\hat{X}}_{k/k-1}\right),$$

The mean-square-error array $P_{k}$ of the state estimate ${\hat{X}}_{k}$ can be expressed as follows:

(14) $$P_{k}=E\left[{\tilde{X}}_{k}{\tilde{X}}_{k}^{T}\right]=\left(I-K_{k}H_{k}\right)P_{k/k-1}{\left(I-K_{k}H_{k}\right)}^{T}+K_{k}R_{k}{K_{k}}^{T},$$

### 2.2. Polynomial Interpolation Theory

Polynomial interpolation belongs to a class of algorithms used in derivative-free optimization methods, which is essentially function approximation. By interpolating a polynomial to a given set of points, a simple and easy-to-compute function is constructed to approximate the objective function, and then the function value of the objective function at a point can be approximated by the function value of the interpolated approximation function at this point. That is, a function *f* is first obtained as a function of values at certain points in the interval [a,b], and then a function *p* is derived from these values by interpolation such that *p* and *f* are very similar [23,24,25,26].

Let y=f(x) be a function defined in the interval [a,b]. It is known that the value of the function of y=f(x) at the n+1 reciprocal points $a\leq x_{0}<\cdots <x_{n}\leq b$ in [a,b] is

(15) $$y_{i}=f\left(x_{i}\right),i=0,\cdots n,$$

For this finite number of points, it is necessary to construct a function y=p(x) that satisfies

(16) $$p\left(x_{i}\right)=y_{i},i=0,\cdots n,$$

Then, p(x) is said to be the interpolating polynomial of the function *f* in the interval [a,b] through the list of points $\{x_{i},y_{i}\},i=0,\cdots n$, where [a,b] is called the interpolating interval, $\{x_{i}\},i=0,\cdots n$ is called the interpolating node, *f* is called the interpolated function, and $f-p_{n}$ is called the interpolating residual term, or error.

Polynomial interpolation is the construction of a polynomial with a degree not exceeding *n*:

(17) $$p_{n}\left(x\right)=a_{0}+a_{1}x+\cdots +a_{n}x^{n},$$

such that it satisfies the interpolation condition $p\left(x_{i}\right)=y_{i},i=0,\cdots n$, i.e.,

(18) $$\left\{\begin{array}{c} a_{0}+a_{1}x_{0}+\cdots +a_{n}{x_{0}}^{n}=f\left(x_{0}\right)\\ a_{0}+a_{1}x_{1}+\cdots +a_{n}{x_{1}}^{n}=f\left(x_{1}\right)\\ \vdots \\ a_{0}+a_{1}x_{n}+\cdots +a_{n}{x_{n}}^{n}=f\left(x_{n}\right) \end{array}\right.,$$

The uniqueness of the existence of a solution to the interpolating polynomial Equation (17) is determined by the uniqueness of the solution to the system of linear equations formed by the coefficients $a_{0},a_{1},...,a_{n}$ in this polynomial. When the determinant of A is not equal to 0, the interpolating polynomial in Equation (17) has a unique solution, and the system of linear equations in Equation (18) has a unique solution, which can be represented as a Vandermonde determinant by the following equation:

(19) $$\left|A\right|=\left|\begin{array}{ccccc} 1 & x_{0} & {x_{0}}^{2} & \cdots & {x_{0}}^{n}\\ 1 & x_{1} & {x_{1}}^{2} & \cdots & {x_{1}}^{n}\\ \vdots & \vdots & \vdots & & \vdots \\ 1 & x_{n} & {x_{n}}^{2} & \cdots & {x_{n}}^{n} \end{array}\right|,$$

When i≠j and $x_{i}\neq x_{j}$, then $\left|A\right|\neq 0$, at which point the equation has a unique solution, and there exists a unique nth-degree polynomial that satisfies the interpolation condition.

For the above polynomial interpolation, one can construct the polynomial $l_{i}\left(x\right)$ with a number not exceeding *n* such that it satisfies

(20) $$l_{i}\left(x_{j}\right)=\delta_{i,j}=\left\{\begin{array}{c} 1,i=j\\ 0,i\neq j \end{array}\right.,$$

From Equation (20), we can see that $x_{j}\left(i\neq j\right)$ is a root of $l_{i}\left(x\right)$ and $l_{i}\left(x_{i}\right)=1$, so that

(21) $$l_{i}\left(x\right)=\frac{\left(x-x_{0}\right)\left(x-x_{1}\right)\cdots \left(x-x_{i-1}\right)\left(x-x_{i+1}\right)\cdots \left(x-x_{n}\right)}{\left(x_{i}-x_{0}\right)\left(x_{i}-x_{1}\right)\cdots \left(x_{i}-x_{i-1}\right)\left(x_{i}-x_{i+1}\right)\cdots \left(x_{i}-x_{n}\right)},$$

Equation (21) can be written in the following form:

(22) $$L_{n}\left(x\right)=y_{0}l_{0}\left(x\right)+y_{1}l_{1}\left(x\right)+\cdots +y_{n}l_{n}\left(x\right),$$

It can be verified that Equation (22) satisfies the interpolation condition by calling $L_{n}\left(x\right)$ the nth Lagrange interpolation polynomial of f(x) and $l_{i}\left(x\right)\left(i=1,2,\cdots n\right)$ the *n*th interpolation basis function, denoted by

(23) $$\omega \left(x\right)=\prod_{i=0}^{n}\left(x-x_{i}\right),$$

Then, there is

(24) $$\omega^{\prime }\left(x\right)=\prod_{j=0,j\neq i}^{n}\left(x_{i}-x_{j}\right),$$

Then, $l_{i}\left(x\right)$ can be expressed as

(25) $$l_{i}\left(x\right)=\frac{\omega (x)}{\left(x-x_{i}\right)\omega^{\prime }\left(x_{i}\right)},$$

Thus, the Lagrange interpolation function is expressed as

(26) $$L_{n}\left(x\right)=\sum_{i=0}^{n}\frac{\omega (x)}{\left(x-x_{i}\right)\omega^{\prime }\left(x_{i}\right)}f\left(x_{i}\right),$$

The interpolating polynomial $L_{n}\left(x\right)$ is an approximation of the interpolated function f(x) that is exact at the nodes but has an error beyond the nodes, $f\left(x\right)-L_{n}\left(x\right)$, which is called the interpolation residual, also known as the truncation error, and is denoted by $R_{n}\left(x\right)$.

For a high-dimensional function using a higher number of polynomials for approximation, the fitting effect is better than when using a lower number of polynomials, but the need to use the interpolation nodes will increase with the increasing degree; in this case, there will be a surge in the amount of computation, and the solution time will increase. To ensure that the navigation system solution is provided in real time, this paper adopts the form of the 4th-order interpolation polynomial.

For the ordinary use of higher-order interpolating polynomials based on equidistant nodes to approximate the Longe function, the interpolating polynomials will have obvious oscillations at the ends of the approximation interval, so it is traditionally believed that it is not appropriate to use higher-order polynomials with equidistant nodes to approximate the Longe function. However, the virtual measurements generated by the simulations in this paper are based on the solved SINS results, and the GPS data are very close to the solved SINS results and thus do not show the Longe phenomenon.

In summary, in this paper, the interpolating polynomial functions of velocity and position are constructed based on the values of the velocity and position of the SINS corresponding to the *k*th moment and the four previous moments, k−1, k−2, k−3, and k−4, assuming that the velocities and positions of the SINS corresponding to the five moments are, respectively,

(27) $$v_{i}=\left[\begin{array}{c} v_{e_{i}}\\ v_{n_{i}}\\ v_{u_{i}} \end{array}\right]\left(i=k,k-1,k-2,k-3,k-4\right),$$

(28) $$p_{i}=\left[\begin{array}{c} L_{i}\\ \lambda_{i}\\ h_{i} \end{array}\right]\left(i=k,k-1,k-2,k-3,k-4\right),$$

Based on the values of velocity and position at these five moments, the 4th-order interpolating polynomial function of velocity $L_{4}\left(v\right)$ and the 4th-order interpolating polynomial function of position are used. $L_{4}\left(p\right)$ can be constructed as follows:

(29) $$\left\{\begin{array}{c} L_{4}\left(v\right)=g\left(SINS_{v_{k-4}},SINS_{v_{k-3}},SINS_{v_{k-2}},SINS_{v_{k-1}},SINS_{v_{k}}\right)\\ L_{4}\left(p\right)=g\left(SINS_{p_{k-4}},SINS_{p_{k-3}},SINS_{p_{k-2}},SINS_{p_{k-1}},SINS_{p_{k}}\right) \end{array}\right.,$$

The approximate GPS velocity and position measures at the *k*th moment can then be obtained by Taylor expansion. This constructs the virtual measurements and also avoids the occurrence of high-order polynomial functions and the Longe phenomenon.

### 2.3. Taylor Expansion Theory Section

In order to facilitate the study of more complex functions, researchers often want to use simple functions to approximate these expressions. Because the polynomial representation of a function only needs to carry out a finite number of addition, subtraction, and multiplication operations on the independent variable to find the value of the function, we often use polynomials to approximate functions. It is natural to think of using higher-order polynomials to approximate functions in order to improve accuracy [27,28,29,30].

According to Taylor’s Median Theorem, if a function f(x) has an nth-order derivative at $x_{0}$, then there exists a neighborhood of $x_{0}$, and for any *x* in that neighborhood, there are the following equations:

(30) $$f\left(x\right)=f\left(x_{0}\right)+f^{\prime }\left(x_{0}\right)\left(x-x_{0}\right)+\frac{f^{\prime \prime }\left(x_{0}\right)}{2!}{\left(x-x_{0}\right)}^{2}+\cdots +\frac{f^{n}\left(x_{0}\right)}{n!}{\left(x-x_{0}\right)}^{n}+R_{n}\left(x\right),$$

(31) $$R_{n}\left(x\right)=o\left({\left(x-x_{0}\right)}^{n}\right),$$

Equation (30) is called the nth-order Taylor polynomial of the function f(x) at $x_{0}$, Equation (31) is called the nth-order Taylor formula of f(x) at $x_{0}$ with a Peano cosubject, and $R_{n}\left(x\right)=o\left({\left(x-x_{0}\right)}^{n}\right)$ is called the Peano cosubject, which is the nth-order Taylor polynomial that approximates the error produced by f(x). This error is infinitesimal when $x\to x_{0}$ is of higher order than ${\left(x-x_{0}\right)}^{n}$. However, it is not possible to estimate the magnitude of the error from it.

For the polynomial interpolation function obtained in Equation (29), a Taylor expansion is performed at the *k*th moment so that three virtual velocity GPS measurement formulas and three virtual position measurement formulas are obtained at the *k*th moment.

(32) $$\left\{\begin{array}{c} f\left(v\right)=f\left(v_{k}\right)+f^{\prime }\left(v_{k}\right)\left(v-v_{k}\right)+\frac{f^{\prime \prime }\left(v_{k}\right)}{2!}{\left(v-v_{k}\right)}^{2}+\frac{f^{\left(3\right)}\left(v_{k}\right)}{3!}{\left(v-v_{k}\right)}^{3}\\ f\left(p\right)=f\left(p_{k}\right)+f^{\prime }\left(p_{k}\right)\left(p-p_{k}\right)+\frac{f^{\prime \prime }\left(p_{k}\right)}{2!}{\left(p-p_{k}\right)}^{2}+\frac{f^{\left(3\right)}\left(p_{k}\right)}{3!}{\left(p-p_{k}\right)}^{3} \end{array}\right.,$$

Bringing *k* into play, the virtual measurement at the *k*th moment is found, i.e.,

(33) $$\left\{\begin{array}{c} Z_{v_{k}}=SINS_{v_{k}}-f\left(v_{k}\right)\\ Z_{p_{k}}=SINS_{p_{k}}-f\left(p_{k}\right) \end{array}\right.,$$

Equation (33) compensates for the significant impact of GPS signal interruptions on the system and avoids the continuous degradation of the estimation accuracy of the relevant parameters, as well as improving the stability of the system.

### 2.4. An Overview of the PIT-AKF Algorithm

When the GPS signal is interrupted, then we can construct a polynomial interpolation dataset based on the value of the SINS, construct the interpolating polynomial functions of velocity and position based on the data in the dataset, simulate the GPS measurement value at the *k*th moment, use the Taylor expansion to find the expressions of the measurement functions of the velocity and position at the *k*th moment, bring *k* in to find the virtual measurement value of the *k*th moment, and then finally substitute the obtained virtual measurement value into the filter calculation.

The following is an expression of the basic steps of the PIT-AKF algorithm developed in this study:

1. Calculate the one-step prediction of the state at moment *k*, i.e., calculate ${\hat{X}}_{k/k-1}$.
2. Judge whether the GPS signal is interrupted; go to 3 if interrupted and to 2 otherwise.
3. Construct polynomial interpolation functions $L_{4}\left(v\right)$ and $L_{4}\left(p\right)$ based on the SINS values solved at moment *k* and the previous four moments.
4. Perform a Taylor expansion of the two polynomial interpolating functions to obtain f(v) and f(p).
5. Substitute f(v) and f(p) at moment *k* to obtain virtual measurements $Z_{v}$ and $Z_{p}$ at moment *k*.
6. Input the obtained virtual measurements $Z_{v}$ and $Z_{p}$ into the filtering equation to compute the new interest $v_{k}=Z_{k}-H_{k}{\hat{X}}_{k|k-1}$.
7. Calculate the one-step prediction mean square error $P_{k/k-1}$.
8. Calculate the gain matrix $K_{k}$.
9. Solve the new filtered solution ${\hat{X}}_{k}$.
10. Solve for the new state estimate mean square error $P_{k}$.
11. Make k=k+1, and return to 1.

**Figure 1:**
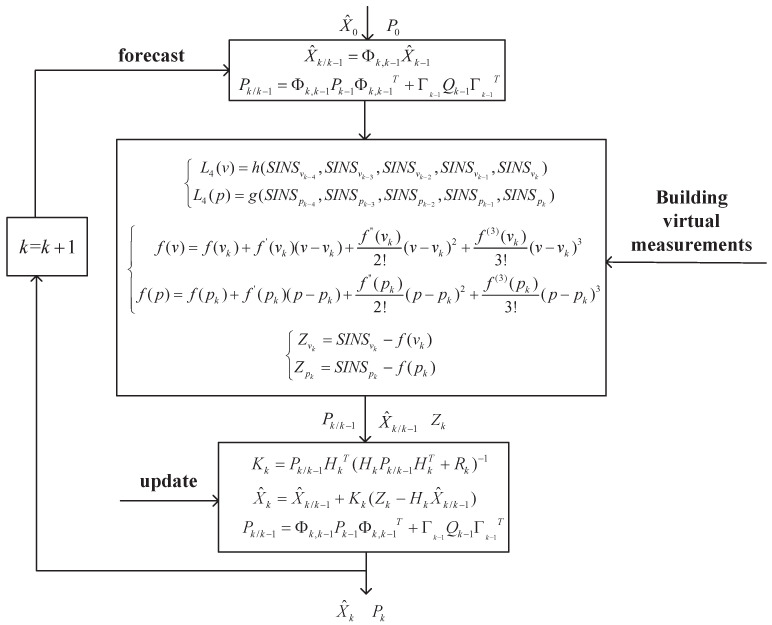
PIT-AKF block diagram.

Figure 1 represents the schematic block diagram of the PIT-AKF algorithm proposed in this paper.

### 2.5. Loosely Combined SINS/GPS Navigation System Modeling

In this paper, the error in the output parameters of the navigation subsystem is used as the state quantity of the system [31]. The idea of the loose combination is to use the available navigation information output from the GPS receiver and the corresponding navigation information after the SINS solution. Then, the difference in navigation information between the two outputs is determined, and the difference is used as the measurement input for the data fusion of the whole combined system. The error model of the SINS is used as the system equation for the data fusion; the error of the SINS is estimated by the data fusion algorithm, and it is corrected. The navigation information here is mainly position and velocity.

In this paper, we propose to use the 18-dimensional SINS solution error values to establish the state equations of the system, which are expressed as follows [32]:

(34) $$\dot{X}\;\left(t\right)=F\left(t\right)X\left(t\right)+G\left(t\right)W\left(t\right),$$

(35) $$X\left(t\right)={\left[\phi_{E}\;\phi_{N}\;\phi_{U}\;\delta_{VE}\;\delta_{VN}\;\delta_{VU}\;\delta L\;\delta \lambda \;\delta h\;\varepsilon_{bx}\;\varepsilon_{by}\;\varepsilon_{bz}\;\nabla_{x}\;\nabla_{y}\;\nabla_{z}\;lever_{x}\;lever_{y}\;lever_{z}\right]}^{T},$$

where ϕ denotes the error estimate for attitude, $\delta_{V}$ denotes the error estimate for velocity, δL denotes the error estimate for latitude, δλ denotes the error estimate for longitude, δh denotes the error estimate for altitude, $\varepsilon_{b}$ denotes gyro zero bias, *∇* denotes accelerometer drift, and lever denotes the spatial rod arm error. In Equation (34), F(t) denotes the state transfer matrix of the system, and G(t) denotes the noise matrix of the system.

The system noise vector is

(36) $$W\left(t\right)={\left[\omega_{gx}\;\omega_{gy}\;\omega_{gz}\;\omega_{ax}\;\omega_{ay}\;\omega_{az}\right]}^{T},$$

In Equation (36), $\omega_{g}$ denotes the random wander error of the gyro, and $\omega_{a}$ denotes the random wander error of the accelerometer.

In this paper, the difference between the values of velocity and position solved by the SINS and the values of velocity and position of the carrier measured by the GPS is used as a volume measure, and the volume measure can be expressed as follows:

(37) $$Z\left(t\right)=\left[\begin{array}{c} V_{E}-V_{EGPS}\\ V_{N}-V_{NGPS}\\ V_{U}-V_{UGPS}\\ L-L_{GPS}\\ \lambda -\lambda_{GPS}\\ h-h_{GPS} \end{array}\right]=\left[\begin{array}{c} \delta_{V_{x}}\\ \delta_{V_{y}}\\ \delta_{V_{z}}\\ \delta L\\ \delta \lambda \\ \delta h \end{array}\right]=H\left(t\right)X\left(t\right)+V\left(t\right),$$

In Equation (37), Z(t), H(t), and V(t) denote the measurement value, measurement matrix, and measurement noise of the integrated navigation system, respectively.

(38) $$\left\{\begin{array}{c} H\left(t\right)=\left[\begin{array}{c} H_{V}\\ H_{P} \end{array}\right]\\ V\left(t\right)=\left[\begin{array}{c} V_{V}\\ V_{P} \end{array}\right] \end{array}\right.,$$

In Equation (38), $H_{V}$ and $H_{P}$ are the velocity and position measurement matrices, respectively, and $V_{V}$ and $V_{P}$ are the GPS velocity and position measurement white noise, respectively.

In summary, the system equations for the GPS/SINS

pine combination in this paper can be obtained as

(39) $$\left\{\begin{array}{c} \dot{X}\;\left(t\right)=F\left(t\right)X\left(t\right)+G\left(t\right)W\left(t\right)\\ Z(t)=H(t)X(t)+V(t) \end{array}\right.,$$

The system equations and the measurement equations can be expressed as shown in Equation (39). Together with the initial values of the required navigation parameters for the filtered solution, the solution can be obtained in accordance with the basic equations of the filtered solution (Equations (1)–(14)), and the estimated values of each navigation parameter and the corresponding covariance matrices can be obtained in the end.

### 2.6. Assessment Indicators and Compared Methods

In this article, we measure the accuracy of navigation parameters by the average root-mean-square error (ARMSE) of each navigation parameter in the integrated SINS/GPS navigation system, with smaller values of ARMSE representing the higher accuracy of the navigation parameters.

By comparing the ARMSE values of the navigation parameters using the three filtering algorithms, it is possible to determine which of the filtering algorithms has a higher accuracy, which means that the estimation accuracy of the relevant navigation parameters and the localization accuracy are higher.

ARMSE is defined as follows

(40) $$\left\{\begin{array}{c} ARMSE_{A}≜\frac{1}{t}\sqrt{E(\left(X_{k}^{A}-{\hat{X}}_{k}^{A}\right){\left(X_{k}^{A}-{\hat{X}}_{k}^{A}\right)}^{T})}\\ ARMSE_{V}≜\frac{1}{t}\sqrt{E(\left(X_{k}^{v}-{\hat{X}}_{k}^{v}\right){\left(X_{k}^{v}-{\hat{X}}_{k}^{v}\right)}^{T})}\\ ARMSE_{P}≜\frac{1}{t}\sqrt{E(\left(X_{k}^{s}-{\hat{X}}_{k}^{s}\right){\left(X_{k}^{s}-{\hat{X}}_{k}^{s}\right)}^{T})} \end{array}\right.,$$

In Equation (40), $ARMSE_{A}$ denotes the attitude mean rms difference, $ARMSE_{V}$ denotes the velocity mean rms difference, $ARMSE_{P}$ denotes the position mean rms difference, and *t* denotes the total time of carrier motion. $X_{k}^{A}$, $X_{k}^{v}$, and $X_{k}^{s}$ denote the three-axis real attitude value, real velocity value, and real position coordinates of the carrier at moment *k*, respectively. ${\hat{X}}_{k}^{A}$, ${\hat{X}}_{k}^{v}$, and ${\hat{X}}_{k}^{s}$ denote the three-axis estimated attitude value, estimated velocity value, and estimated position coordinates of the carrier at moment *k*, respectively [32].

## 3. Results

We designed a computer simulation experiment and a real road test to illustrate the performance advantages and disadvantages of the three filtering algorithms, PIT-AKF, UMI-KF, and SKF, in the integrated SINS/GPS navigation experiment.

### 3.1. Simulation Experimental Design and Experimental Results

The computer simulation experiment is divided into two parts, which are the combined SINS/GPS navigation experiment during measurement interruption in the segmented motion state and the combined SINS/GPS navigation experiment during measurement interruption in the full-motion state.

For the computer simulation experiments, we simulated the situation where the carrier encounters the GPS signal interruption while moving in the computer and simulated the carrier making various complex motions, which in turn simulated various common motions of a carrier in the real world. At this point, we compared the estimation accuracies of velocity, position, and attitude obtained by our proposed algorithm (PIT-AKF) with the filtering algorithm without any measures (UMI-KF) and the standard Kalman filtering algorithm (SKF), which measured the normal situation in this paper’s experiments. Thus, we can compare the advantages and disadvantages of the different solutions, and the advantages and disadvantages of the different algorithms are also contrasted. Some experimental conditions set up in the computer are given below, including the experimental parameters of the trajectory simulated by the computer and some parameters of the GPS and IMU outputs.

#### 3.1.1. Parametric Design of the Trajectory Generator Section

The following modes of motion of the object simulated in the computer are stationary, accelerated motion, uniform motion, decelerated motion, left-turning motion, and right-turning motion. These, in turn, simulate the various common ways in which carriers move in the real world.

For the segmented motion state, the acceleration, uniform speed, turning, and deceleration times were set to 60 s each, the signal interruption time was set to 20 s each, and the GPS signal interruption time accounted for 33% of the carrier’s segmented motion time. The settings of the parameters of the trajectory generator in the segmented motion state are no longer given, and the design of the parameters of the GPS and IMU part is consistent with the parameter settings in the full motion.

For the full-motion state, the GPS signal interruption time was set to vary from 5 to 30 s, the interruption time of all analog GPSs was 180 s, the total time of carrier motion was set to 720 s, and the interruption time of the GPS signal accounted for 25% of the whole carrier motion time. The parameter setting of the trajectory generator part and the parameter design of the GPS and IMU part in the full-motion state are given below.

In order to observe the trajectory of the carrier more clearly, the simulated trajectory diagram and velocity change diagram of the carrier in full motion are given below. From the trajectory diagram, it can be seen that the carrier has carried out straight-line motion, left-turn motion, right-turn motion, and straight-line motion. From the velocity change diagram, it can be seen that the carrier has experienced a stationary state, uniformly accelerated motion, uniformly accelerated motion, uniformly decelerated motion, and uniform motion.

**Figure 2:**
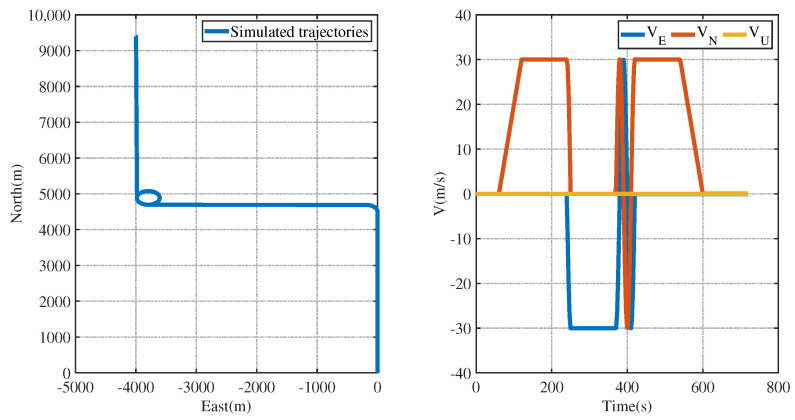
Mode of trajectory and velocity change of the carrier in full-motion state.

Figure 2 represents the motion trajectory modal map and velocity change map of the carrier in the full motion state.

Table 1 represents some of the parameters of the trajectory generator at full motion. They have the same values in the three experiments.

#### 3.1.2. GPS and IMU Partial Parameter Design

In the experiments, it was vital to make sure that the GPS and IMU data under all three algorithms, SKF, UMI-KF, and PIT-AKF, were the same so that the advantages and disadvantages of the different algorithms could be compared. Some of the experimental parameters are given below; they were set the same in all three experiments.

Table 2 represents some performance reference values for GPS and IMU.

#### 3.1.3. Simulation Experiment Results

The simulation plots of the RMSE values with respect to time are given below for the simulated carrier segmented motion and full-motion states.

**Figure 3:**
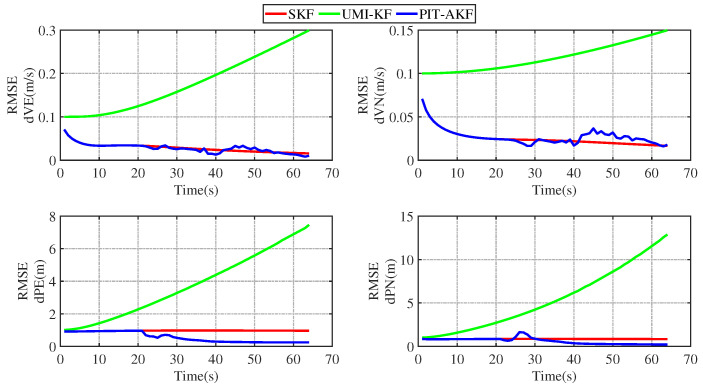
Simulation of velocity and position RMSE values in segmental acceleration state.

**Figure 4:**
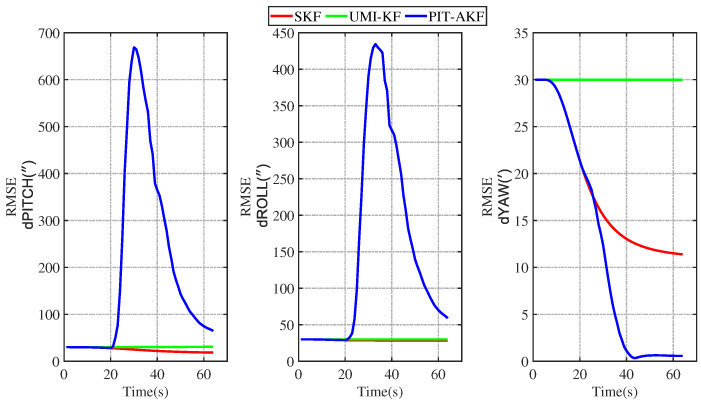
Simulation of attitude angle RMSE values in segmented acceleration state.

From Figure 3 and Figure 4, it can be seen that the estimation errors of the carrier’s velocity, position, and attitude are all stabilized within the normal range and gradually converge and stabilize, indicating that the PIT-AKF algorithm proposed in this paper can work well in the accelerated state when the carrier faces a GPS interruption and effectively suppresses the divergence of the individual navigation parameters.

**Figure 5:**
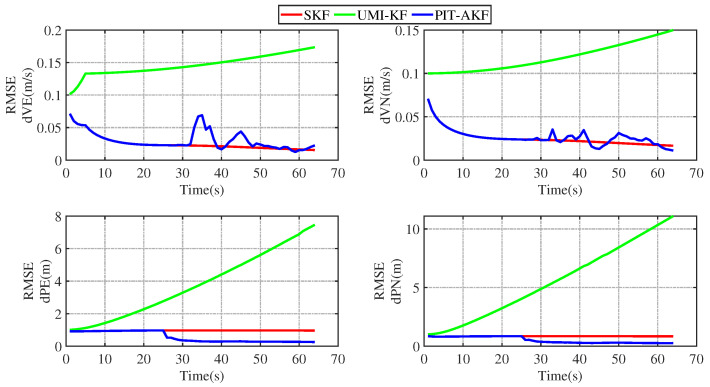
Simulation of velocity and position RMSE values in segmented uniform velocity state.

**Figure 6:**
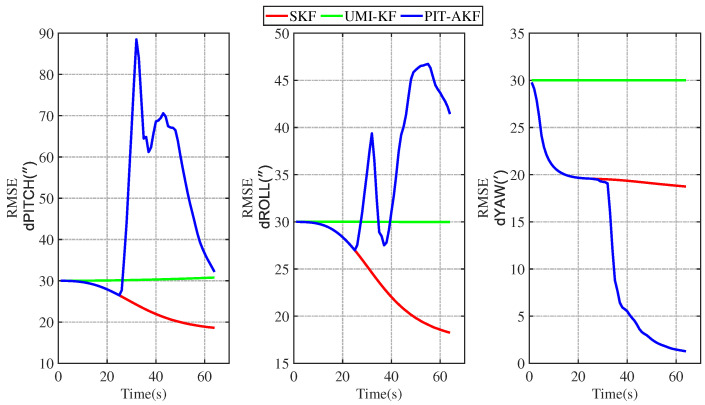
Simulation of RMSE value of attitude angle at segmented uniform velocity state.

In Figure 5 and Figure 6, it can be seen that the estimation errors of the carrier’s velocity, position, and attitude are all stabilized within the normal range and gradually converge and stabilize, indicating that the PIT-AKF algorithm proposed in this paper can work well when the carrier faces GPS interruptions at a uniform speed, effectively suppressing the dispersion of each navigation parameter.

**Figure 7:**
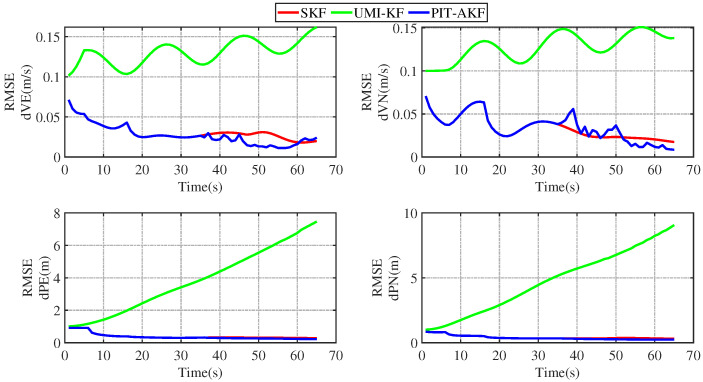
Simulation of velocity and position RMSE values in segmented turning condition.

**Figure 8:**
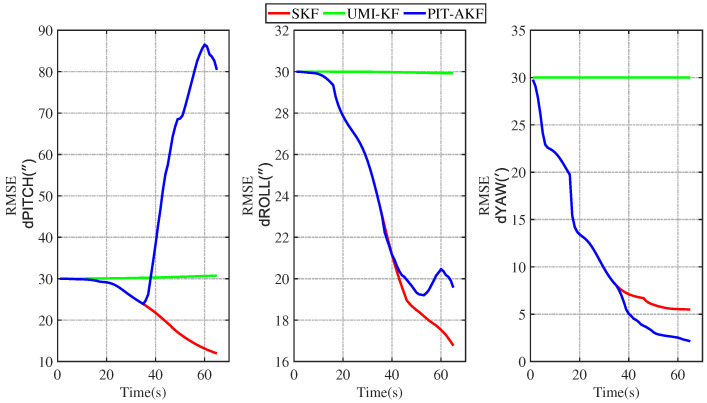
Simulation of attitude angle RMSE values in segmented turning condition.

As can be seen in Figure 7 and Figure 8, the estimation errors of the carrier’s velocity, position, and attitude are all stabilized within the normal range and gradually converge and stabilize, indicating that the PIT-AKF algorithm proposed in this paper can work well when the carrier faces a GPS interruption in the turning state, effectively suppressing the divergence of each navigation parameter.

**Figure 9:**
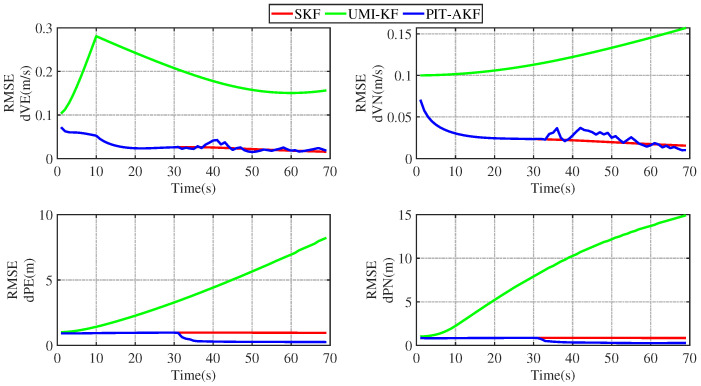
Simulation of velocity and position RMSE values in segmental deceleration state.

**Figure 10:**
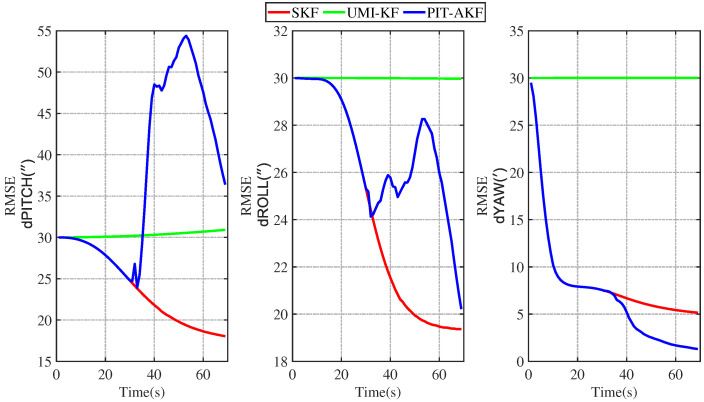
Simulation of attitude angle RMSE value in segmental deceleration state.

As can be seen in Figure 9 and Figure 10, the estimation errors of the carrier’s velocity, position, and attitude are all stabilized within the normal range and gradually converge and stabilize, indicating that the PIT-AKF algorithm proposed in this paper can work well in the decelerated state when the carrier faces a GPS interruption, which effectively suppresses the dispersion of each navigation parameter.

**Figure 11:**
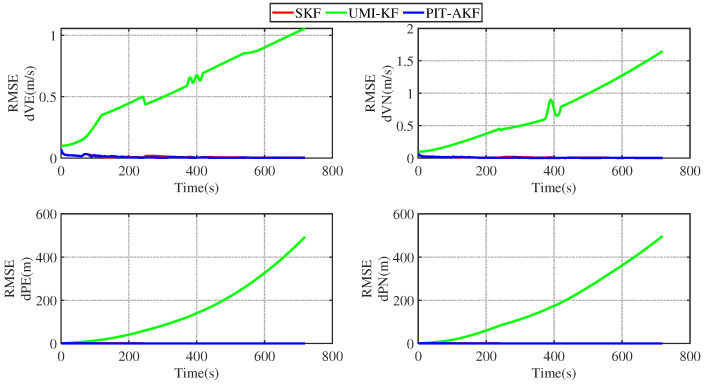
Simulation of velocity and position RMSE values in full-motion state.

**Figure 12:**
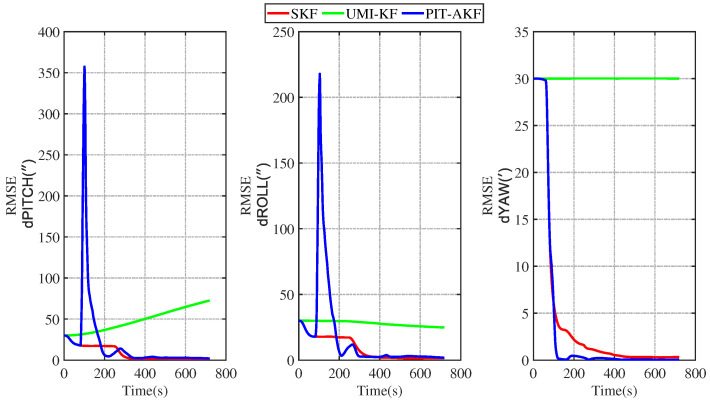
Simulation of RMSE values of attitude angle in full-motion state.

In Figure 11 and Figure 12, it can be seen that the estimation errors of the carrier’s velocity, position, and attitude are all stabilized within the normal range and gradually converge and tend to be stable, indicating that the PIT-AKF algorithm proposed in this paper can work well when the carrier faces a GPS interruption in the full-motion state and effectively suppresses the divergence of each navigation parameter.

### 3.2. Experimental Design and Results of Roadway Field Tests

**Figure 13:**
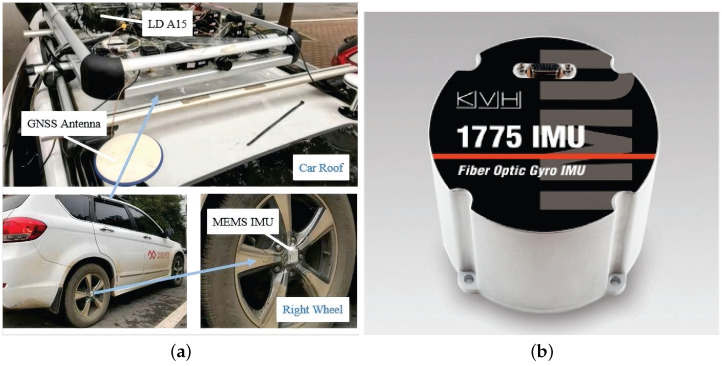
Schematic diagram of data acquisition for the integrated SINS/GPS navigation system. (**a**) represents a similar analog diagram for collecting actual road measurement data, (**b**) represents a schematic of the shape of the inertial measurement unit.

In order to fully evaluate the accuracy of the algorithm proposed in this paper (PIT-AKF) for the SINS/GPS loose combination navigation system when there is an interruption of the gauge information in the carrier, we conducted a road test with real data, which was provided by a laboratory of Wuhan University and downloaded from www.psins.org.cn, accessed on 15 October 2023, at the website. A ground vehicle equipped with a GPS-IMU device in an urban environment was used. A large amount of IMU and GPS data were collected. The schematic diagram of the equipment installation for data collection was similar to that shown in Figure 13; the antenna was placed on the roof of the vehicle to collect signals from global navigation satellites, and the inertial navigation unit was placed at the center of the right rear tire axle to collect IMU data. The data were collected for a total of 5300 s, including the output data of the IMU, the velocity and position output data of the GPS, and the attitude reference output, velocity reference output, and position reference output processed by the IE software. The data processed by the IE software were used as the true values for comparison.

As shown in Figure 13, the 1775 IMU is a commercial off-the-shelf (COTS) inertial measurement unit provided by KVH. The 1775 IMU is designed to provide high-level measurement performance and is capable of working in challenging environments. The 1775 IMU is equipped with a 25 g accelerometer, with dimensions of 88.9 mm (diameter) × 73.7 mm (height), making it ideal for highly dynamic applications and applications with high acceleration, vibration, or impact levels.

Table 3 represents the list of IMU and GPS partial parameters used in the actual road measurements.

In order to simulate the scenario where the carrier has a measurement signal interruption, 720 s of experimental data were intercepted. The GPS signal interruption time was set to vary from 5 to 30 s every 45 s or so, and the simulated GPS interruption time was 180 s. The total carrier motion time was 720 s, and the GPS signal interruption time accounted for 25% of the entire carrier motion time.

#### Experimental Results of Road Measurement

Some images of the carrier during real road movement in the face of the interruption of the measured signal are given below. These are plots of the real trajectory and the velocity over time of the carrier’s movement on the road, as well as estimated root-mean-square error (RMSE) comparison plots of each navigation parameter under different algorithms, and finally, a table of the average root-mean-square error (ARMSE) comparisons of the navigation parameters under different filtering algorithms is also given.

**Figure 14:**
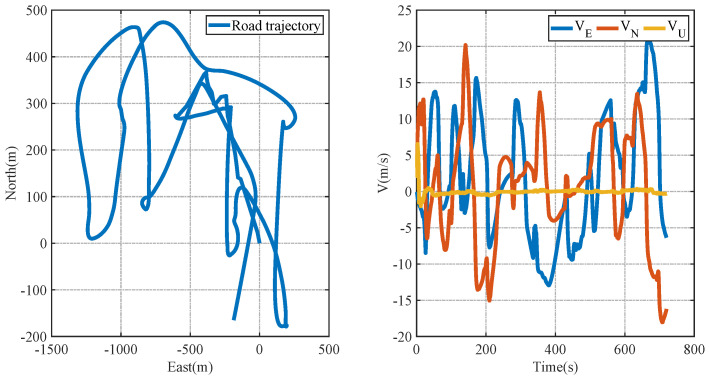
Carrier trajectory map and velocity changes under real road conditions.

Figure 14 represents the carrier trajectory map and velocity change map under roadway real-world conditions.

## 4. Discussion

### 4.1. Analysis of Simulation Experiment Results

Figure 2, Figure 3, Figure 4, Figure 5, Figure 6, Figure 7, Figure 8, Figure 9, Figure 10, Figure 11 and Figure 12 represent the trend plots of velocity and position rms error values as well as attitude rms error values of the carrier over time in segmental acceleration, segmental uniform velocity, segmental turning, segmental deceleration, and full-motion states, respectively. As can be seen from the above image, at the moment when the GPS signal is interrupted, the measurement information of the whole combined SINS/GPS navigation system is missing. The estimation error of each navigation parameter of the carrier will have some fluctuation at the moment of the missing measurement information.

From the above figure, it can be seen that the assignment of each navigation parameter under the UMI-KF algorithm fluctuates the most. This is because when the GPS signal is interrupted, the GPS cannot provide effective speed and position information, resulting in the inability to provide accurate measurement information. At this time, the entire filtering and solving process is left with only the state update, but not the measurement update, and the combined SINS/GPS navigation mode degrades to a purely inertial guidance system, which will lead to the accumulation of navigation errors and thus the dispersion of each of the carrier’s navigation parameters. And under the action of the SKF algorithm, at this time, the measured information is only the solved value of the inertial guidance system, which will lead to the measured information sharply increasing, and it introduce a large error into the filtering and solving processes, so each navigation parameter appears to have a certain dispersion.

Although the amplitude of each navigation parameter under our proposed PIT-AKF algorithm also varies with certain ups and downs, the amplitude is smaller and the convergence time of the estimation error of each navigation parameter is shorter. This indicates that the virtual measurement constructed by polynomial interpolation and Taylor expansion in this paper can well compensate for the impact of the lack of measurement information on the integrated SINS/GPS navigation system, reflecting higher integrated navigation estimation accuracy and stronger system stability, but the estimation accuracy of each navigation parameter is slightly lower than that of the SKF algorithm when the measurement is not interrupted.

### 4.2. Analysis of the Results of the Real Road Test

**Figure 15:**
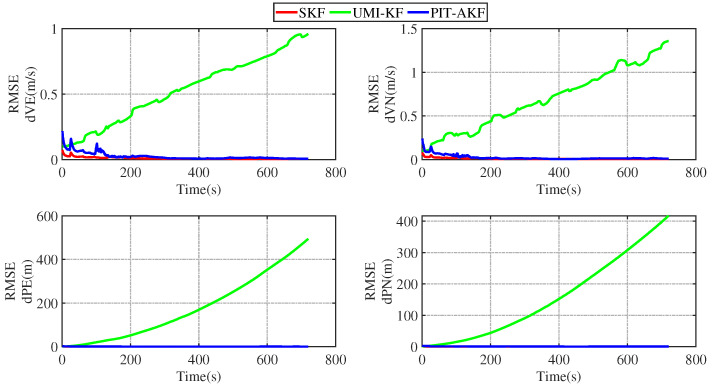
Simulation of speed and position RMSE values under real road conditions.

**Figure 16:**
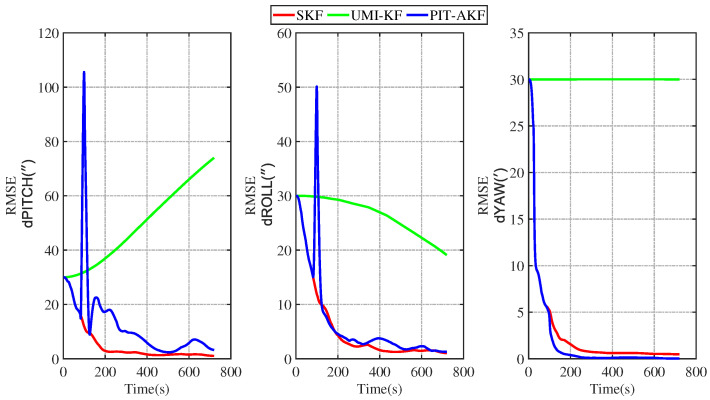
Simulation of attitude angle RMSE values under road real conditions.

Figure 15 and Figure 16 represent the trend plots of the velocity and position rms error values and attitude rms error values of the carrier over time under real road test conditions. As can be seen from the above figures, the real road test once again illustrates the effectiveness and superiority of the PIT-AKF algorithm.

From Table 4 above, it can be concluded that when the carrier experiences a perturbation due to a measurement signal interruption, our proposed PIT-AKF filtering algorithm outperforms the UMI-KF algorithm without any measures in terms of the carrier’s three-axis attitude estimation accuracy, velocity estimation accuracy, and positional localization accuracy. The experimental results based on the measured data of the loosely combined SINS/GPS navigation system show that when the carrier is in the interference environment with interrupted measurement signals, our proposed PIT-AKF solution embodies higher navigation accuracy, higher positioning accuracy, and stronger system reliability. Compared with the UMI-KF algorithm without any measures, the average estimation error accuracies of the three-axis attitude angle, the average velocity estimation error accuracies in the eastward and northward directions, and the average position estimation error accuracies in the eastward and northward directions with the PIT-AKF filtering algorithm proposed in this paper are improved substantially, and they are close to the estimation accuracies of the measurements in normal conditions. As can be seen from the contents of the above table, the effectiveness, high accuracy, and strong robustness of the PIT-AKF solution proposed in this paper are proved once again.

Both simulation experiments and real road measurement experiments demonstrate the superior performance of our proposed PIT-AKF solution at the moment of measurement interruption. At the moment of measurement interruption, the virtual measurement volume is constructed quickly through polynomial interpolation and Taylor expansion, which ensures the measurement update, avoids the degradation of the combined SINS/GPS navigation method into a purely inertial navigation system, and avoids the accumulation of navigation parameter errors and the dispersion of the localization information; thus, it can provide high-precision navigation information.

## 5. Conclusions

Aiming at the problem of GPS signal interruptions faced by carriers in the loosely combined SINS/GPS navigation system, we propose a Kalman filtering algorithm based on polynomial interpolation and Taylor expansion in this paper. By constructing a polynomial interpolation function based on the velocity and position information solved by the SINS during the period of the measurement signal interruption and then using a Taylor expansion of the function to construct a virtual measurement, we effectively make up for the significant impacts of the lack of measurement information on the navigation system. Our proposed PIT-AKF solution is proved in simulation and real data experiments of the navigation system. By constructing the polynomial function and Taylor expansion, the algorithm proposed in this paper improves the estimation accuracy of the relevant parameters of the navigation system and the carrier’s localization accuracy and enhances the robustness of the system.

## Figures and Tables

**Table 1:** Partial parameter settings of the trajectory generator in the full-motion state.

Motion State	Static	Average Acceleration	Uniform Velocity	Turn Left	Uniform Velocity	Turn Right	Uniform Velocity	Uniform Deceleration	Uniform Velocity	Company
Carrier movement time	60	60	120	10	120	50	120	60	120	(s)
GPS signal interruption time	0	20	30	5	30	15	30	20	30	(s)
Total duration					720					(s)
SINS solution cycle					0.01					(s)
Filter cycle					1					(s)
Initial value of attitude angle					(0,0,0)					(°)
Initial value of velocity					(0,0,0)					(m/s)
Initial value of position	Longitude: 108.909664 (east), Latitude: 34.246048 (north)									(°)

**Table 2:** GPS and IMU performance reference.

Meaning	Experimental Value	Company
Gyro wander	[0.03;0.03;0.03]	(°/h)
Accelerometer zero offset	[100;100;100]	(μg)
Randomized gyro angle wandering	[0.001;0.001;0.001]	°/$\sqrt{h}\;$
Random accelerometer speed wandering	[10;10;10]	μg/$\sqrt{\mathrm{Hz}}$
Measurement noise vectors for GPS noise	(0.1,1)	(m/s,m)
Frequency of IMU	100	(Hz)
Setting frequency of GPS	1	(Hz)

**Table 3:** The selected IMU and GPS parameters used in the measurements.

IMU	1775 IMU Parameter Value
INS output frequency	100 Hz
Gyro range	$490^{{}^{\circ}}$/s
Accelerometer range	±25 g
Gyro bias stability	$\pm 0.5^{{}^{\circ}}$/h
Accelerometer bias stability	±0.25 mg
Velocity random walk	≤0.12 mg/Hz
Angle random walk	$\leq 0.12^{{}^{\circ}}$/h
GPS output frequency	1 Hz

**Table 4:** Measuring the ARMSE values of navigation parameters under each Kalman filtering algorithm in an interrupted environment.

Navigation Parameters	SKF	UMI-KF	PIT-AKF
ARMSE-dPITCH ${(}^{{}^{\circ}})$	0.00148	0.01368	0.00357
ARMSE-dROLL ${(}^{{}^{\circ}})$	0.00149	0.00734	0.00187
ARMSE-dYAW ${(}^{{}^{\circ}})$	0.04372	0.50006	0.03329
ARMSE-dVE (m/s)	0.00982	0.53180	0.00804
ARMSE-dVN (m/s)	0.01006	0.69542	0.00770
ARMSE-dPE (m/s)	0.23762	1.76521	0.20376
ARMSE-dPN (m/s)	0.25601	1.54589	0.22744

## Data Availability

The data used to support the finding of this study are available from the corresponding author upon request.

## Abbreviations

The following abbreviations are used in this manuscript:

SINS	Strapdown Inertial Navigation System
GPS	Global Positioning System
RMS	Root-mean-square
RMSE	Root-mean-square error
2D-LDV	2D Laser Doppler Velocimeter
UWB	Ultra-Wide Band
VIO	Visual–Inertial Odometry
ARMSE	Average root-mean-square error
PIT-AKF	An adaptive Kalman filtering algorithm for measurement interruption
	based on polynomial interpolation and Taylor expansion
SKF	Standard Kalman filter algorithm
UMI-KF	Kalman filtering algorithm without any measures in the face of a
	measurement interruption
IMU	Inertial measurement unit
COTS	Commercial off-the-shelf
RMSE-DA	Root-mean-square error of attitude angle estimation
RMSE-dV	Root-mean-square error of velocity estimation
RMSE-dP	Root-mean-square error of position estimation
